# Forest structure determines nest box use by Central European boreal owls

**DOI:** 10.1038/s41598-022-08792-y

**Published:** 2022-03-18

**Authors:** Richard Ševčík, Bohuslav Kloubec, Jan Riegert, Jiří Šindelář, Marek Kouba, Markéta Zárybnická

**Affiliations:** 1 Faculty of Environmental Sciences, Czech University of Life Sciences Prague, Kamýcká 129, 165 00 Praha – Suchdol, Czech Republic; 2 Faculty of Science, University of South Bohemia, Branišovská 1760, 370 05 České Budějovice, Czech Republic; 3 Faculty of Agrobiology, Food and Natural Resources, Czech University of Life Sciences Prague, Kamýcká 129, 165 00 Praha – Suchdol, Czech Republic

**Keywords:** Behavioural ecology, Conservation biology, Population dynamics, Behavioural ecology, Conservation biology, Population dynamics, Animal behaviour

## Abstract

Nest boxes represent a popular tool to support secondary cavity-nesting species. Surprisingly, the benefits and limitations of nest boxes for target species in different environments are poorly understood. We performed a 3-years experimental study in two different Central European forests to evaluate nest box use and breeding performance of boreal owl (*Aegolius funereus*) — a species well known for its readiness to occupy nest boxes. Based on territorial vocalisation, two boreal owl populations 200 km apart were similarly abundant in their environments. However, only the boreal owl population in young restored Norway (*Picea abies*) and blue (*Picea pungens*) spruce-dominated forests on mountain plateaus readily occupied nest boxes with the occupancy reaching 8–15%. Nest boxes lost their supporting function for the boreal owl in mature Scots pine (*Pinus sylvestris*)-dominated forests in the lowland, where the nest box occupancy reached 0–1%. As a result, the population of boreal owls that used nest boxes in the young restored forests produced 10 times more fledglings than the population inhabiting mature Scots pine forests. We explain the differences by the contrasting availability of natural tree cavities between the two study areas being much higher in mature Scots pine forests. For the first time, this study documents differences in nest box use despite similar food availability and population size of the target species. The study provides the findings-related recommendations for deploying nest boxes for boreal owls and points out a general lack of practical guides.

## Introduction

The availability of natural tree cavities is crucial for survival of secondary cavity-nesting species, including owls^1,2^. For example, the boreal owl, *Aegolius funereus,* depends on the availability of cavities excavated by the black woodpecker, *Dryocopus martius*^3,4^. Such cavities occur more frequently in management-free, old deciduous, and pine forests than in spruce monocultures^5–7^. When natural cavities are rare or lacking, artificial opportunities may play a key role for secondary-cavity nesters. Nest boxes are a case of a worldwide popular tool to enhance the availability of nest sites^2,8,9^. They also allow studying the breeding and trophic ecology of species, their life-history strategies, interspecific interactions, and provide conditions for camera nest monitoring that would be hard to do in natural cavities (e.g.,^3,9–11^). However, the readiness to use nest boxes differs among species (e.g., boreal owl vs. pygmy owl *Glaucidium passerinum*^4^), and it can even vary within one species under variable environments (e.g., great tit *Parus major*^12^). Despite the high popularity of nest boxes among amateur and professional ecologists, studies comparing the effectiveness of nest boxes in different environments and providing practical guides for deploying nest boxes for specific species are often entirely lacking.

The boreal owl is a secondary cavity nester with a Holarctic distribution, spreading across the boreal forest of northern North America, Europe, and Asia^13^. In Europe, this threatened species (European directive 2009/147/EC, Annex I) exhibits foraging, habitat, and nest-site specialization^14^. It predominantly inhabits coniferous forests in the northern latitudes and coniferous or mixed forests in high altitudes in Central and Southern Europe^4,15^. This species is limited by the availability of natural tree cavities excavated by black woodpeckers; however, it also readily breeds in artificial wooden boxes (e.g.,^3,16^). The readiness of this species to occupy nest boxes makes this owl a ‘textbook example’ of a species whose breeding biology and trophic ecology have been primarily discovered based on nest box populations (e.g.,^3,17,18^). However, the nest box occupancy by this species varies hugely over regions, countries, and continents, reaching from units to tens of percentages (Table 1). Surprisingly, we still poorly understand the general pattern of the nest box occupancy by boreal owls and the breeding performance of this species under various environments (for rare study, see^19^).

**Table 1:** Nest box use by the boreal owl in Europe, North America, and Asia; the state, locality, elevation, period, the number of boxes, nests, box-years, and the nest box occupancy (expressed as the proportion of occupied nest boxes of checked boxes). The way how authors described their results varied hugely. Therefore, we present the information on the number of boxes, nests, and nest box occupancy from long-term studies either as a sum counted for the entire study period (marked as ‘Total’) or a yearly mean (marked as ‘Mean’) with minimum (‘Min’) and maximum (‘Max’) values.

State	Locality	Elevation	Period	No. of boxes/ year	No. of box-years	No. of nests			Nest box occupancy (%)				References
						Total	Min	Max	Total	Mean	Min	Max	
Canada	Southern Yukon		1984–96	13–105	573	6			1				^20^
Canada	Alberta region	470–920	2016	169	169	4			2				^21^
USA	Alaska	90–150	1995–97	36	108	29			27				^22^
USA	Alaska	200–650	2005–06	91, 108	199	23			12				^23^
USA	Alaska	110–690	2015	200	200	27			14				^24^
USA	Rocky Mts.	1700	1985–88	45	180	3			2				^17^
USA	Idaho	1520–2140	1988–90	283–450	1016					4			^25^
USA	Rocky Mts.		1995–01	250–450						1			^26^
China	Lianhuashan Mts.		2003–07	67	335		4	7			6	10	^27^
Sweden	Västerbotten		1980–84	500	2500	525	4	201	21		1	40	^28^
Sweden	Västerbotten		1981–82, 1984–85	44–149		330	5	99		30	6	66	^29^
Sweden	Västerbotten		1998–99	300	600	84					15	22	^30^
Sweden	Västerbotten		2006–07	273, 275	548	47			9		2	15	^31^
Finland	Kauhava region	30–120	1966–85	35–450	4577	352	2	63	8		2	22	^32^
Finland	Kauhava region	30–120	1966–08	hundreds	677	104			15				^3^
Czech Rep.	Ore Mountains	730–960	2000–03	100	400	72	10	26	18		10	26	^33^
Czech Rep.	Šumava Mts.	500–1300	1984–05	211	4448	316			7				^34^
Czech Rep.	Šumava Mts.	500–1100	1992–02	395		250				6	4	8	^35^
Czech Rep.	Šumava Mts.	400–1378	1978–02		5006	299				6			^36^
Czech Rep.	Krkonoše Mts.		1985–86	40, 60	100	1			1		0	2.5	^37^
Switzerland, France	Jura Mts.	1000–1600	1985–14	64–116	2550	425	2	39	17		3	55	^38^
Germany	Kaufunger Wald	250–580	1965–84	60	1200	76			6				^39^
Germany	Harz Mts.	450–850	1979–91	250	3250	390			12				^40^
Germany	Olpe	430–580	1981–10	4–64	1034	187	0	20	18		0	56	^41^
Italy	Cansiglio Highland		1989–20	80–100	2400	93			4				^42^
Serbia	Kopaonik National Park	800–2017	2011–13	63	189	9			5				^43^

We performed nest box and territorial vocal experiments in two contrasting environments in Central Europe (Czech Republic) to examine the use of nest boxes and their benefits for target species, using boreal owl as a case species. We expected i) to find a higher nest box occupancy by boreal owls in young restored Norway (*Picea abies*) and blue (*Picea pungens*) spruce-dominated forests than in Scots pine (*Pinus sylvestris*) forests due to the substantially increased availability of natural cavities in the second area. As a result, ii) the fledgling productivity of the boreal owl population using nest boxes would be higher in young restored forests than in mature forests. We also expected to find iii) a higher nest box occupancy by other secondary cavity-nesting birds (i.e., passerines) in young restored Norway and blue spruce-dominated forests than in Scots pine forests and iv) no evidence for an interspecific competition limiting nest box use by boreal owls in any study area. Finally, we aimed to use our findings to create specific recommendations for deploying nest boxes for the boreal owl.

## Materials and methods

### Study areas

**Figure 1:**
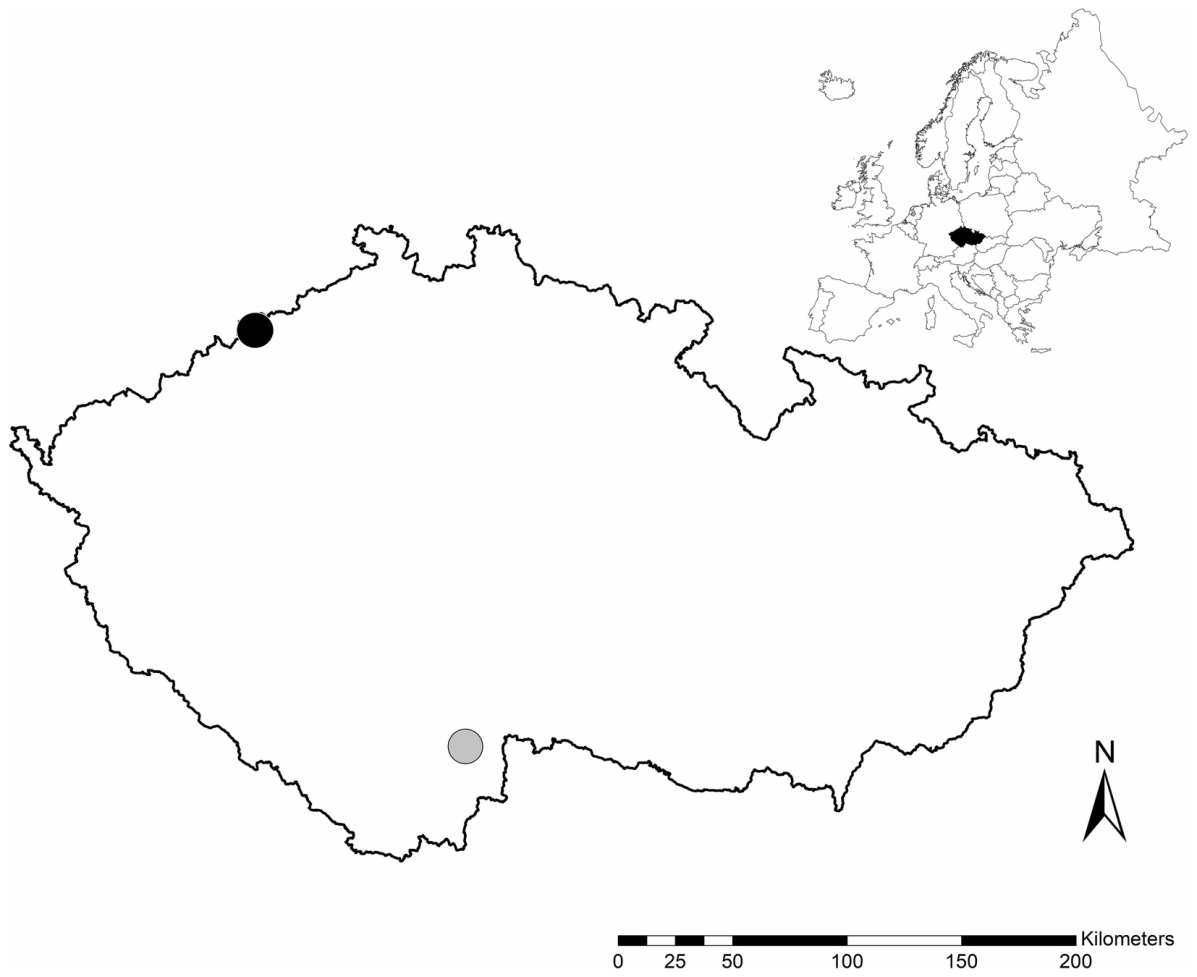
Two study areas in the Czech Republic: a historically air-polluted area in the Ore Mts. (51°N, 14°E, black point) and a protected landscape area in the Trebon Basin (49°N, 15°E; grey point).

In 2015–2017, we conducted the experimental study in two environments, in the northwest and the southern Czech Republic (200 km apart; Fig. 1), which differed in forest structure, elevation, and climate conditions (Table 2). We located the first study area in a historically air-polluted area on the Ore Mountains plateau, neighbouring Saxony, Germany. We have been using this study area for the boreal owl nest box research since 1999^33,44^. We gradually erected the second study area in 2012–2014 in the lowland Protected Landscape Area of the Trebon Basin close to the border to Austria.

**Table 2:** Basic information about the study areas. Geographical position, the size of the area, elevation, climate conditions (mean daily temperature, mean daily rainfall, and the yearly mean number of days with continual snow cover), habitat composition (based on CORINE Land Cover 2018), and estimates of the number of black woodpecker’s territories (per 1 km^2^) and tree cavities (per 1 ha) excavated by black woodpeckers are shown.

	Ore Mts.			Trebon Basin		
		%	Mean ± SD		%	Mean ± SD
Latitude, longitude	51°N, 14°E			49°N, 15°E		
Area (km^2^)	150			400		
Elevation (m a. s. l.)	700–920		791.8 ± 69.8	420–500		456.9 ± 19.6
Daily temperature (°C)			7.0 ± 0.3			9.2 ± 0.6
Daily rainfall (mm)			2.5 ± 0.3			1.5 ± 0.2
Snow cover (days)			80.3 ± 17.6			31.7 ± 20.4
Forest habitat		87.0			47.0	
Pasture and grasslands		9.6			15.0	
Agriculture area		1.9			13.0	
Water area		1.0			10.0	
Urban area		0.5			15.0	
No. of territories	0.5–1			1–10		
No. of tree cavities	0–1			1–5		

The two study areas differed in the history of human activities. The Ore Mts. were exposed to extreme SO_2_ and NO_x_ pollution emitted in the 1970–80s from factories located in the foothills, followed by extensive forest losses, dramatic changes in animal communities, and massive restoration processes hindered by acid soil, harsh mountain climate, and extensive damage to young plantations caused by cervids^45^. Nowadays, the habitat of the Ore Mts. is formed by a mosaic of large areas of young coniferous (mainly native Norway spruce and non-native blue spruce) and deciduous stands, small patches (usually 0.5–2.0 ha) of mature Norway spruce, and old solitary European beech (*Fagus sylvatica*) trees. The Trebon Basin has been a protected landscape area since 1979, accompanied by forest, water, and agriculture management. The habitat consists of mature Scots pine and Norway spruce coniferous forests formed as monocultures with numbers of small (usually less 1-ha) clear-cuts.

### Forest structure comparison

**Figure 2:**
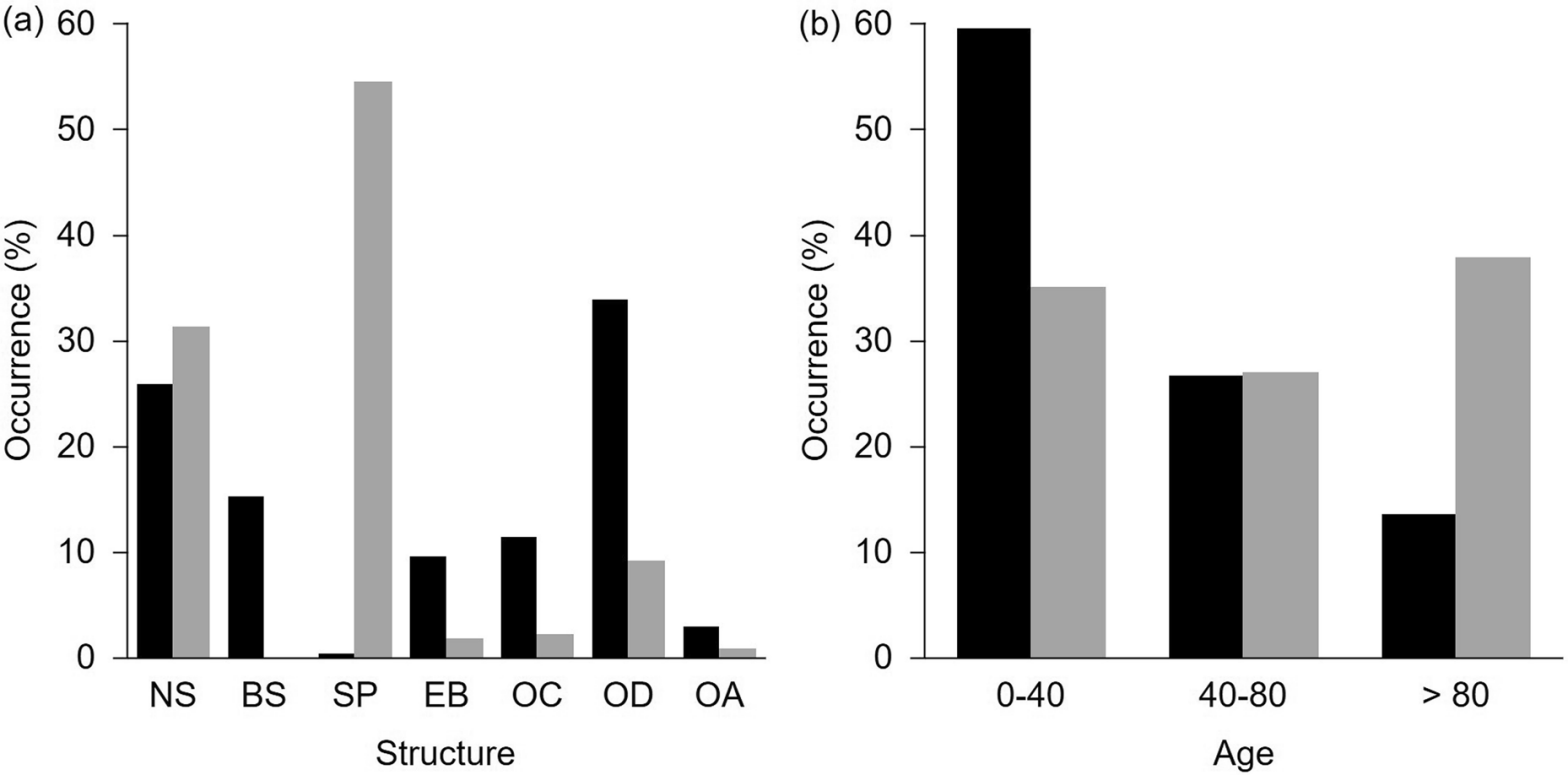
The structure (**a**) and age (**b**) of forest stands (in proportions) in the Ore Mts. (black bars) and the Trebon Basin (grey bars). NS – Norway spruce, BS – blue spruce, SP – Scots pine, EB – European beech, OC – other coniferous species, OD – other deciduous species, OA – open areas. Data are evaluated based on the vegetation maps of the Czech Forestry Institute.

Forest habitats dominated both study areas, covering 87% and 47% of the Ore Mts. and Trebon Basin study area, respectively. The area of any of the other habitat types (pasture and grasslands, agriculture, water, and urban areas) did not exceed 15% (for details, see Table 2; data based on CORINE Land Cover 2018). We used vegetation maps of forest stands (the Czech Forestry Institute, 2015–2017) to compare species and age forest structures between the study areas. First, we grouped forest stands into seven categories according to the dominant tree species (i.e., Norway spruce, blue spruce, Scots pine, European beech, other coniferous stands, other deciduous stands, and clear-cuts). We counted the proportion of each species category within each study area. We found that the structure of forest stands differed significantly between the areas (Chi-squared test: Chi = 722.59, *P* < 0.001, df = 6). Norway (26.0%) and blue (15.4%) spruces were dominant tree species in the Ore Mountains, while Scots pine (54.5%) and Norway spruce (31.3%) were dominant tree species in the Trebon Basin (Fig. 2a). Second, we grouped forest stands into three categories according to age (0–40 years, 40–80 years, > 80 years). We counted the proportion of each age category within each study area. We found the age of forest stands significantly differed between the areas (Chi-squared test: Chi = 32.67, *P* < 0.001, df = 2). The less-than-40 years habitats dominated in the Ore Mts. On the contrary, all age categories of forest stands in the Trebon Basin occurred evenly (Fig. 2b).

### Black woodpecker populations

We recorded a substantially more abundant population of black woodpeckers (assessed based on territorial calls^46^) and number of cavities excavated by this species in the Trebon Basin than the Ore Mts. (Table 2).

### Nest box occupancy

To evaluate nest box occupancy, we used an existing nest box system in the Ore Mts. (for details, see the chapter Study areas) in which 246 nest boxes were evenly distributed in forest habitats over an area of 150 km^2^ with a density (mean ± SD) 1.65 ± 0.05 nest boxes/km^2^. In 2012–2014, we erected a similar nest-box distribution scheme in the Trebon Basin. Forest habitats of this area were separated by other habitat types (i.e., pastures, agriculture, water, and urban areas) to a greater extent than in the Ore Mts., covering a total of 400 km^2^. In the Trebon Basin, we evenly distributed 245 nest boxes with a density (mean ± SD) of 0.60 ± 0.01 nest boxes/km^2^.

We provided the same type of nest boxes in both study areas. The nest boxes were made of 2-cm thick wooden planks. The bottom area dimensions counted for 20–25 × 20–25 cm, the height of the walls was 40 cm, and the roof exceeded the front wall by 5 cm. The entrance had a diameter of 8 cm. All nest boxes were painted with dark brown colour and filled with a 3–5 cm layer of sawdust, resulting in an effective distance of 20–22 cm from the bottom (i.e., top of the sawdust) to the lowest part of the entrance. We positioned nest boxes 3–5 m above the ground and kept them in good conditions, i.e., they were repaired, cleaned, and filled with new sawdust after each breeding season. The surrounding of all nest box entrances was kept free of vegetation. The mean age of nest boxes (i.e., the number of years since the nest box installation or reinstallation counted for all nest boxes in all study years) was (mean ± SD) 4.9 ± 4.4 years in the Ore Mts. (n = 722 boxes) and 3.2 ± 1.1 years in the Trebon Basin (n = 724 boxes).

We identified nest box use by boreal owls and other animals based on present animals and their active nests or reliable traces of animal activities (e.g., an abandoned nest or nest material) inside the boxes. Each year, we checked 230–246 boxes in the Ore Mts. and 237–245 boxes in the Trebon Basin to identify the number of boreal owl nests. We included nest boxes with the presence or absence (0/1) of boreal owl nests in the GLMM analysis (see below). We further conducted synchronized spring (i.e., in April 2015 and May 2016–2017) and autumn (in September 2016 and October 2017) nest box inspections in both study areas to identify nest box use by boreal owls and other animal taxa. We synchronously checked 230–246 nest boxes (i.e., 230, 246, and 246 boxes in both spring and autumn) and 56–245 nest boxes (i.e., 245, 242, and 237 boxes in spring and 0, 56, and 200 boxes in autumn) during spring and autumn nest box inspections in 2015–2017 in the Ore Mts. and Trebon Basin, respectively. We included nest boxes with the presence or absence (0/1) of other taxa in the CCA analysis (see below). We also calculated the nest box occupancy rate as the number of boxes with present animal taxa or their traces per all checked nest boxes within a specific period and year. Alternatively, we presented the nest box occupancy as the proportion of occupied nest boxes of all erected nest boxes.

### Boreal owl nests

We accomplished additional inspections in the boxes where boreal owl nests occurred to determine the number of eggs, fledglings, nests depredated by martens (*Martes* sp.), and nests abandoned by boreal owl females with no fledgling produced (according to^19^). We counted boreal owl breeding productivity based on these data; however, we did not include additional inspections in statistical analyses.

### Owls’ vocalisation

At the beginning of April and May in 2015–2017 (a total of six recording events), we distributed audio recordings across both study areas to monitor the presence and absence of all owl species based on territorial vocal activity. In the Ore Mts., we placed 36 audio recorders during each recording event on sampling points within a regular grid with a span of (mean ± SD) 2.0 ± 0.3 km (min–max: 1.6–2.6 km, 25–75% range: 1.8–2.1 km, see also^47^). In Trebon Basin, we placed 32 audio recorders within a span of 2.5 ± 0.6 km (min–max: 1.7–3.4 km, 25–75% range: 1.9–2.7 km). We installed audio recorders (Olympus DM650, Olympus Corporation, Japan) with automatic time-recording settings during suitable weather conditions (i.e., without strong wind and precipitations) on tree trunks at the height of 1.5 m above the ground. We left audio recorders exposed for one night during each recording event (April, May 2015–2017). We set the recording time of each audio recorder (at least) from 8 PM to 6 AM, allowing us to evaluate owl vocalisation during continuous 10-h recording at each sampling point (recorder) and recording event. We collected a total of 216 and 192 recordings (sampling points) in the Ore Mts. and Trebon Basin, respectively, out of which we excluded 13 and 14 recordings due to technical failures. As a result, we used a total of 2030 h (i.e., 203 sampling points) in the Ore Mts. and 1780 h (i.e., 178 sampling points) in the Trebon Basin to evaluate territorial owl calls.

We transformed particular audio recordings into spectrograms and analysed them using AMSrv software^48^, setting the spectrum at 1 min with FFT length, the window size of 4096 × 1366. We used no filters to remove background noise. We recognized the vocal activities of owls based on territorial calls of particular species^46^. We included sampling points with vocal presence or absence (0/1) of each owl species into subsequent analyses. Simultaneously, we calculated the vocal occupancy rate as the number of sampling points with the vocal presence of owl species per all sampling points, sorted according to the sampling period and year. Based on vocal activity, we also estimated the density for each owl species per 10 km^2^, considering the area of 3.14 km^2^ (radius 1 km) per one sampling point.

### Small mammal abundances

The boreal owl primarily feeds on small mammals, mainly *Microtus* and *Myodes* voles, *Apodemus* mice, and *Sorex* shrews^18,49^. The abundance of voles and mice affects breeding characteristics, including territorial vocal activity^47^, breeding performance^19^, home range size^50^, and parental investment^51^. Therefore, we assessed the abundance of small mammals using snap trapping carried out every year at the beginning of June by setting up snap traps at six blocks (30 × 90 m, 4 × 10 traps, 10-m span) within representative habitats of each study area. The traps were left for three days in the field and checked every morning. We determined all captured small mammals (n = 102 individuals) to species and grouped them into four categories: *Apodemus* mice, *Microtus* voles, *Myodes* voles, and *Sorex* shrews. We also calculated the abundance index as the number of captured individuals of each category per 100 trap nights for each trapping site (for details, see^18^).

### Statistical analyses

We used generalized linear mixed models (GLMM) in R 4.0.2 software^52^ to assess the effect of the study area and year on the abundance of small mammals. Using *lmer* function (package Lme4), we created four models with *Apodemus* mouse, *Myodes*, and *Microtus* vole, and *Sorex* shrew abundances as dependent variables. We calculated the number of trapped individuals per 100 trap nights for each trapping site, study area, and year (trapping index) for each dependent variable. The study area, year, and the interaction of the study area and year were used as independent variables and the trapping site as a random effect. We also calculated partial relationships between the study areas and years using a post-hoc test (function *lsmeans* in lsmeans package).

To compare the nest box occupancy by boreal owls between the study areas, we used GLMM analysis (*lmer* function) with a binomial distribution of the dependent variable. We examined the effect of the study area on the occurrence of boreal owl nests (0/1). We included the density of nest boxes (i.e., the number of nest boxes within a buffer of 1000 m of each nest box) as a covariate and ID box and year as random effects.

We performed a multivariate canonical correspondence analysis (CCA) in Canoco 5 software^53^ to examine the effect of primary predictors, i.e., the study area, year (2015–2017), and period (April, May) on the vocal occurrence (0/1) of each owl species. We used the sampling point of each audio recording as a categorical covariate. Further, we computed another CCA analysis to examine the effect of primary predictors, i.e., the study area, year (2015–2017), and period (spring, autumn) on nest box occupancy (0/1) by boreal owls and other animal taxa (i.e., pine marten *Martes martes*, passerines, common goldeneye *Bucephala clangula*, insects, bats). We used the number of boxes in a buffer of 1000 m around each box (continuous variable) and ID box (categorical variable) as covariates.

### Ethical statement

We conducted our research in conformance with all applicable laws; we worked under the permissions of SR/0006/TR/2015 and 173/049/ZPZ/2015/ZD-838 issued by the Nature Conservation Agency of the Czech Republic.

## Results

### Small mammal abundances

*Apodemus* mice and *Myodes* voles were the most abundant species in both study areas, counting 54.0% (n = 41) and 40.8% (n = 31) of all trapped individuals in the Ore Mts. and 53.9% (n = 14) and 38.5% (n = 10) of all trapped individuals in the Trebon Basin. *Microtus* voles and *Sorex* shrew were rare species (< 4%) in both study areas (Supplementary Information, Table S1). The abundance of *Myodes* voles, *Microtus* voles, and *Sorex* shrews did not differ between study areas and years (Supplementary Information, Table S2). However, the abundance of *Apodemus* mice was significantly affected by the interaction of the study area and year (GLMM; Chi = 13.45, df = 32, *P* = 0.004, Supplementary Information, Table S2). A post-hoc test showed that *Apodemus* mice were significantly more abundant in 2017 in the Ore Mts. compared to the Trebon Basin (post-hoc test; t = 3.58, *P* = 0.011, Supplementary Information, Table S3. In the Ore Mts., the abundance of *Apodemus* mice also differed significantly between 2016 and 2017 (post-hoc test; t =  − 4.21, *P* = 0.004, Supplementary Information, Table S3).

### Owls’ vocalisation

**Figure 3:**
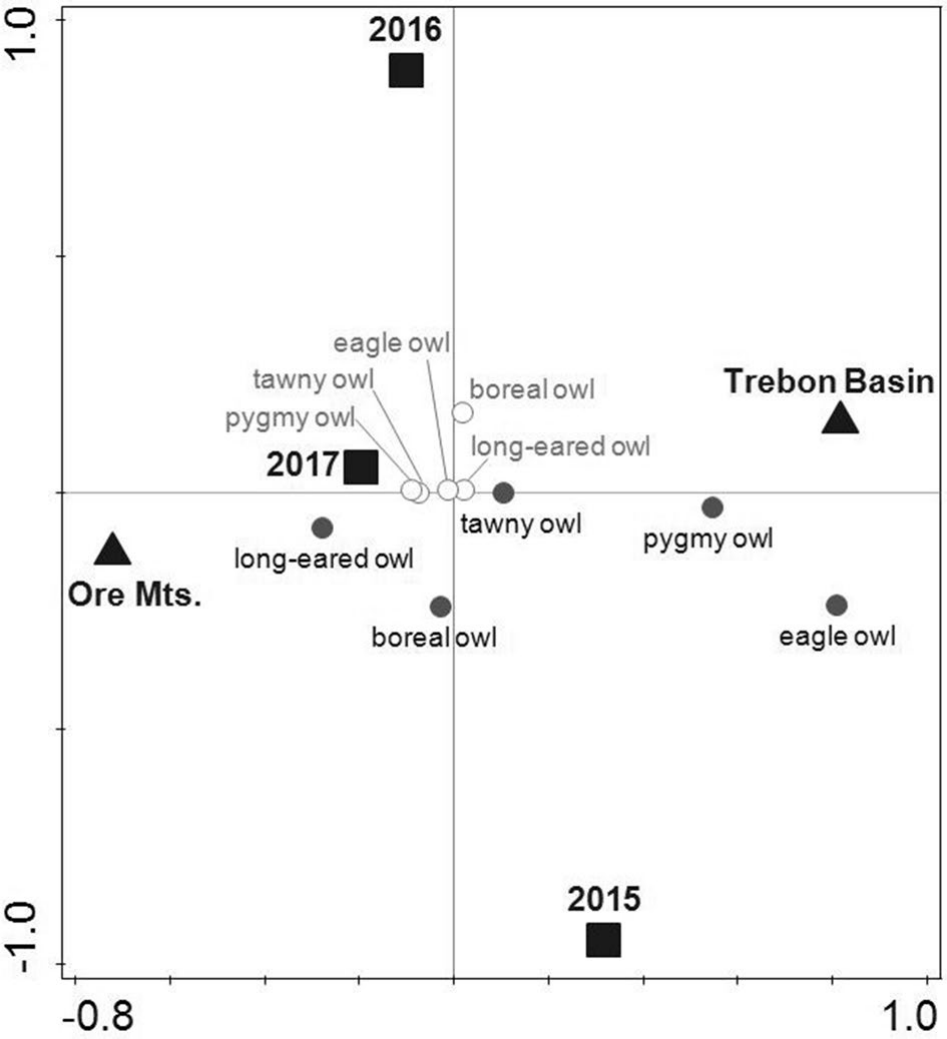
The effect of the study area (Ore Mts. and Trebon Basin, black triangle) and year (2015–2017, black square) on the distribution of owl species (boreal, tawny, pygmy, long-eared, and eagle owls) based on the vocal activity of owls. The presence (grey circle, black front) and the absence (white circle, grey front) of individual owl species are shown. The canonical correspondence analysis (CCA) was used. The first and second ordination axes together explained 94.7% of the variability.

Based on territorial vocal activity, we identified the same five owl species — boreal owl, tawny owl *Strix aluco*, long-eared owl *Asio otus*, Eurasian pygmy owl, and Eurasian eagle owl *Bubo bubo* — within each study area. The vocal occurrence rate of owl species (all owl species included) varied from 0 to 0.59 and 0 to 0.58 per sampling point in the Ore Mts. and Trebon Basin, respectively (Supplementary Information, Table S4). Using CCA, we found a significant effect of the study area and year on the vocal occurrence of owl species, but the sampling period (April, May) had no effect (Table 3). The first and second ordination axes explained together 94.7% of the variability. Boreal and tawny owls were the most frequently recorded owl species in both study areas and all years (Supplementary Information, Table S4). Comparing the study areas showed that the boreal owl vocal occupancy rate was similar and simultaneously the highest in 2015 in both study areas (Fig. 3, Supplementary Information, Table S4). However, it was slightly higher in 2016 and 2017 in the Ore Mts. compared to the Trebon Basin (Fig. 3, Supplementary Information, Table S4). Contrary, the vocal occupancy rate of the tawny owls was similar in 2016 in both study areas, but it was higher in 2015 and 2017 in the Trebon Basin compared to the Ore Mts. (Supplementary Information, Table S4). Pygmy owls occurred more frequently in the Trebon Basin than in the Ore Mts.; however, long-eared owls showed an opposite trend (Fig. 3, Supplementary Information, Table S4). Eagle owl was a rare species in both study areas; it was absent in 2015 and 2017 in the Ore Mts. and 2016 in the Trebon Basin (Fig. 3, Supplementary Information, Table S4).

**Table 3:** The results of CCA analyses. First analysis: the effect of the study area (Ore Mts., Trebon Basin), year (2015–2017), and sampling period (April, May) on the vocal occurrence (0/1) of each owl species. Second analysis: the effect of the study area, year, and sampling period (spring, autumn) on nest box occupancy (0/1) by animal taxa (boreal owl, goldeneye, pine marten, passerines, bats, and insects).

Response variable	Explanatory variable	% of explained variability	F	P
Vocal occupancy rate	Study area	56.53	8.2	0.002
	Year	26.37	3.8	0.002
	Sampling period	8.28	1.2	0.306
Nest box occupancy rate	Study area	36.87	30.0	0.002
	Sampling period	50.31	40.4	0.002
	Year	8.05	6.6	0.002

The density of vocalising owl populations reached yearly 0–1.87 and 0–1.85 individuals per 10 km^2^ in the Ore Mts. and Trebon Basin, respectively (for details, see Supplementary Information, Table S4). Boreal and tawny owls were the most abundant species in the Ore Mts., followed by long-eared owls, pygmy owls, and eagle owls. Tawny and boreal owl were the most abundant species in the Trebon Basin, followed by pygmy owl, long-eared owl, and eagle owl (Supplementary Information, Table S4).

### Boreal owl populations

Boreal owls readily used nest boxes in the Ore Mts., but not in the Trebon Basin. We identified six main differences. First, boreal owls nested in boxes (pooled spring and autumn inspections) more often in the Ore Mts. than the Trebon Basin (GLMM; Chi = 43.27, df = 1441, *P* < 0.001, Table 4). Second, during the spring inspections, the nest box occupancy reached 7–10% in the Ore Mts., but only 0–1% in the Trebon Basin (Supplementary Information, Table S5). Third, the total number of boreal owl nests (both active and abandoned) found during spring and autumn nest box inspections was more than 10 times higher in the Ore Mts. (76 nests) than in the Trebon Basin (5 nests, Table 5). Thus, the total nest box occupancy reached 8–15% in the Ore Mts. and only 0–1% in the Trebon Basin. Fourth, the boreal owl females breeding in nest boxes produced yearly 128.3 ± 49.4 eggs and 63.3 ± 52.1 fledglings (mean ± SD, n = 76 nests) in the Ore Mts., but only 6.0 ± 5.6 eggs and 5.3 ± 4.7 fledglings (n = 5 nests) in the Trebon Basin (Table 5). Fifth, pine martens predated six boreal owl nests in the Ore Mts. and one nest in the Trebon Basin (Table 5). Finally, the boreal owl females abandoned 24 nests in the Ore Mts., and no in the Trebon Basin (Table 5).

**Table 4:** The results of GLMM analysis (*lmer* function). The effect of the study area on nest box occupancy (0/1) by boreal owls.

Model	AIC	df	Chi	% of explained variability	P
Null model	591.88	1442			
Null model + area	550.61	1441	43.27	7.41	< 0.001

**Table 5:** Boreal owls nesting in 2015–2017 in the Ore Mts. and Trebon Basin: the vocal occupancy rate, the density of vocalising individuals, the proportion of spring (i.e., only nests during spring inspections) and total (i.e., all nests) nest box occupancy, the number of boxes, all nests, active nests (i.e., during spring nest box inspections), additional nests (i.e., during autumn nest box inspections), successful nests, nests predated by martens, nests abandoned by boreal owl females (including the proportion of all nests), and the total number of eggs and fledglings.

	Ore Mts.					Trebon Basin				
	2015	2016	2017	Mean ± SD	Mean % ± SD	2015	2016	2017	Mean ± SD	Mean % ± SD
**Vocal occupancy rate**										
April	0.59	0.47	0.50	0.52 ± 0.06		0.52	0.23	0.34	0.36 ± 0.15	
May	0.50	0.17	0.39	0.35 ± 0.17		0.58	0.19	0.34	0.37 ± 0.20	
**Vocal density per 10 km**^**2**^										
April	1.87	1.50	1.59	1.65 ± 0.19		1.64	0.72	1.09	1.15 ± 0.46	
May	1.59	0.55	1.24	1.13 ± 0.53		1.85	0.61	1.09	1.18 ± 0.63	
Spring nest box occupancy (%)	10	9	7	8.7 ± 1.5		1	< 1	0	0.7 ± 0.6	
Total nest box occupancy (%)	15	9	8	10.7 ± 3.8		1	< 1	0	0.7 ± 0.6	
Nest box occupancy rate	0.15	0.09	0.08	0.10 ± 0.04		0.01	< 0.01	0	0.007 ± 0.006	
No. of boxes	230	246	246	240.7 ± 9.2		245	242	237	241.3 ± 4.0	
No. of all nests	34	23	19	25.3 ± 7.7		3	2	0	1.7 ± 1.5	
No. of active nests (spring)	23	23	18	21.3 ± 2.9		3	2	0	1.7 ± 1.5	
No. of additional nests (autumn)	11	0	1	4.0 ± 6.1		0	0	0	0.0	
No. of successful nests	27	6	13	15.3 ± 10.7	58.0 ± 28.2	2	2	0	1.3 ± 1.2	83.3 ± 23.6
No. of predated nests	0	5	1	2.0 ± 2.6	9.0 ± 11.3	1	0	0	0.3 ± 0.6	16.7 ± 23.6
No. of abandoned nests	7	12	5	8.0 ± 3.6	33.0 ± 16.8	0	0	0	0.0	
No. of eggs	181	83	121	128.3 ± 49.4		11	7	0	6.0 ± 5.6	
No. of fledglings	114	10	66	63.3 ± 52.1		9	7	0	5.3 ± 4.7	

### Nest box occupancy by other animals

**Figure 4:**
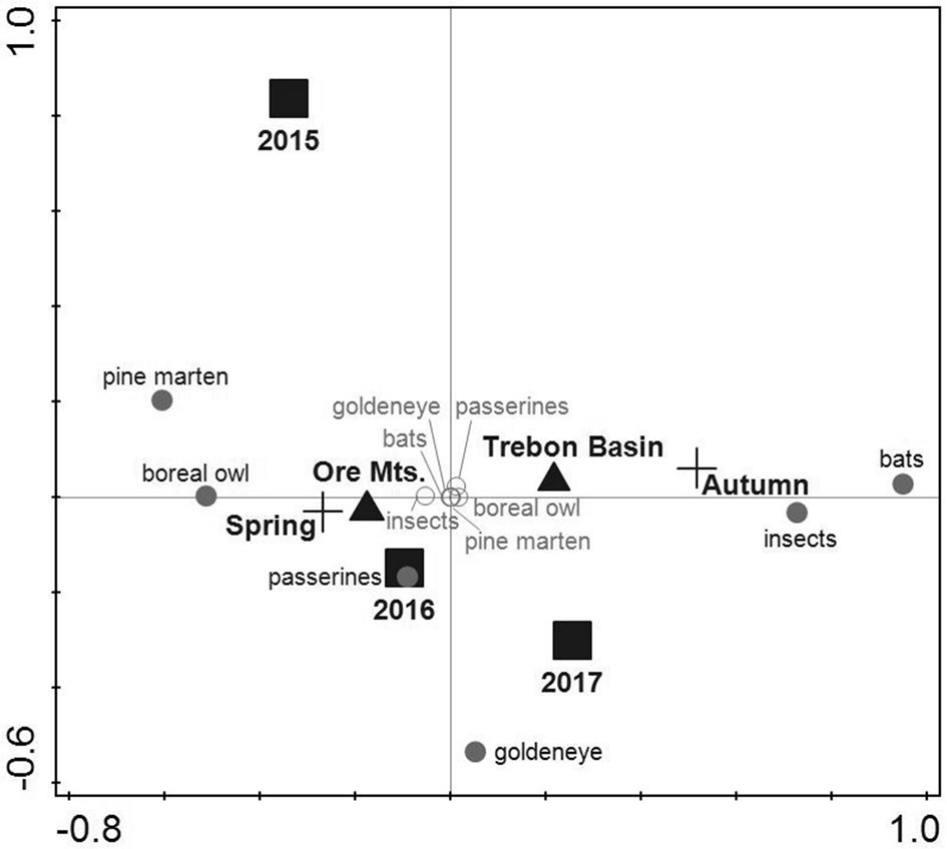
The effect of the study area (Ore Mts. and Trebon Basin, black triangle), year (2015–2017, black square), period (spring and autumn nest box inspections, black cross) on nest box use by different taxa (boreal owl, pine marten, goldeneye, passerines, bats, and insects). The presence (grey circle, black front) and the absence (white circle, grey front) of different taxa are shown. The canonical correspondence analysis (CCA) was used. The first and second ordination axes together explained 98.7% of the variability.

We identified a total of 487 animal activities during synchronized 2,194 spring (April–May) and autumn (September–October) nest box inspections in the two study areas (Supplementary Information, Table S5). Using CCA, we found a significant effect of the study area, year, and sampling period on the occurrence of animal species in nest boxes (Table 3). The first and second ordination axes of the model explained 98.7% of the variability together. Boreal owls, passerine birds, and pine martens were more frequent users in the Ore Mts., while bats, insects, and common goldeneyes occurred more often in the Trebon Basin (Fig. 4).

During the spring (April–May) nest box inspections, passerine birds (Paridae > 90%) and boreal owls were the most frequent dwellers of nest boxes in the Ore Mts. (Fig. 4). Their nest box occupancy reached 5–30% and 7–10%, respectively (for details, see Supplementary Information, Table S5). Pine martens rarely occupied nest boxes in the Ore Mts. during spring (nest box occupancy was ≤ 1%; Supplementary Information, Table S5. The only passerine birds (Paridae > 90%) were occasional spring nesters in the Trebon Basin, reaching 7–8% occupancy. Boreal owls, common goldeneyes, and pine martens were rare spring nesters in the Trebon Basin (nest box occupancy was ≤ 1%; Supplementary Information, Table S5).

During the autumn nest box inspections, no animals inhabited nest boxes in the Ore Mts. Only additional abandoned nests of passerine birds (nest box occupancy reached 6–8%) and one boreal owl nest (< 1%) were built after completing our spring nest box inspections (Supplementary Information, Table S5). Insects (Vespidae) appeared in only one nest box in the Ore Mts. (Supplementary Information, Table S5). On the contrary, insects (Vespidae and Apidae > 90%) were most often dwellers of the nest boxes during autumn inspections in the Trebon Basin (Fig. 4); their occupancy reached 44–52% (Supplementary Information, Table S5). Additional abandoned passerine nests also occurred during the autumn inspections in the Trebon Basin, reaching the occupancy of 0–19% (Supplementary Information, Table S5). Finally, bats occupied nest boxes in this study area (Fig. 4), with less than 2% (Supplementary Information, Table S5).

## Discussion

### Population size and nest box use

Based on territorial vocal activity, we found that the two boreal owl populations in the Ore Mts. and Trebon Basin were of similar size in 2015, and that the one in the Ore Mountains was more abundant in 2016 and 2017 than in the Třeboň Basin. However, the Ore Mts.’ boreal owls used nest boxes much more frequently than their counterparts in the Trebon Basin in all study years; the yearly nest box occupancy reached 8–15% and 0–1%, respectively. As a result, the boreal owl population using nest boxes in the Ore Mts. produced 10 times more fledglings than the same population in the Trebon Basin.

In general, the readiness of boreal owls to use nest boxes is inconsistent over the Holarctic region, reaching the nest box occupancy of 0–66% (for details, see Table 1). Some studies have shown that boreal owls preferred using nest boxes to natural cavities; however, other studies have documented that they did not use nest boxes at all or only rarely. The authors have suggested various reasons for the variability in nest box occupancy rate, including the availability of natural cavities, different breeding success in nest boxes and tree cavities, food availability, and nest box age. Our experimental study has documented that the occupancy rate differed significantly between the two study habitats, even though food abundances and the boreal owl population sizes were comparable. We suggest that the main reason for this contrast lies in the different availability of natural cavities that resulted from different forest structures between the two study areas (the first hypothesis). The explanations are following:

- 1. Availability of natural cavities
  Natural nest-site opportunities for boreal owls are represented almost solely by the cavities excavated by black woodpeckers^3,16^. Their availability increases with increasing age of the forests, and simultaneously, it is higher in deciduous and pine forests than spruce forests^7,54,55^. In our study areas, the species and age forest structure differed substantially. It influenced the abundance of black woodpecker populations and the availability of tree cavities excavated by this species being much higher in mature Scots pine-dominated forests of the Trebon Basin than young restored forests of the Ore Mts. During our long-term study in the Ore Mts. (since 1999; Zárybnická, unpublished data), we recorded even a higher nest box occupancy rate by boreal owls, up to 26% (2001–2006), than in the current study (2015–2017). Over the years, this decrease in a nest box occupancy rate probably resulted from gradual vegetation succession accompanied by the increasing availability of tree cavities excavated by black woodpeckers (Zárybnická and Kilb, unpublished data). However, the abundance of the black woodpecker population and the availability of natural cavities are still much lower in the Ore Mts. than in the Trebon Basin nowadays. Therefore, we suggest that the lack of natural tree cavities was the main reason for a substantially higher readiness of boreal owls to occupy nest boxes in the Ore Mts. Following our third hypothesis, we also found a lower nest box occupancy rate by other secondary cavity-nesting birds, i.e., passerine birds, in the Trebon Basin compared to the Ore Mts. (0–19% vs. 5–30%). Additionally, pygmy owls — which almost solely use natural tree cavities for their breeding and avoid nest boxes in Central European conditions^4^ — were substantially higher in their population size in the Trebon Basin than the Ore Mts. Riegert and Kloubec (unpublished data) have confirmed that pygmy owls frequently nest in natural cavities while avoiding nest boxes in the Trebon Basin.

- 2. Age of nest boxes
  The occupancy rate of nest boxes by boreal owls significantly decreases with their age^3,56^. The increased risk of nest predation by martens in older boxes^57,58^ or food depletion in the vicinity of old nest boxes^59^ have been suggested to explain this effect in boreal owls. However, we found a significantly higher nest box occupancy rate in the Ore Mts. than in the Trebon Basin, even though the mean age of nest boxes (all boxes in all study years) was about 1.7 years higher in the Ore Mts. than in the Trebon Basin. Therefore, the age of nest boxes was not a reason for differences in the nest box occupancy rate between the study areas.

- 3. Maintaining nest boxes
  The quality of nest boxes and their maintenance can affect nest box use by boreal owls. For example, it is essential to install nest boxes high enough above the ground, regularly relocate and maintain nest boxes, keep nest box content dry and clean, and keep the surrounding of the nest box entrance free of branches (Zárybnická, unpublished data). Still, experimental studies are lacking. We used the same quality, maintenance, and installation of nest boxes in both study areas, preventing these factors from consideration as relevant reasons for differences in boreal owls’ nest box occupancy between the study areas.

- 4. Competition with other animals
  Other animals can compete for nest boxes with boreal owls. For example, Hruška^60^ documented two boreal owl nests in natural cavities that failed after wild bees occupied them. Similarly, hornets, wasps, bumblebees^61,62^, or ants (Riegert, unpublished data) can compete with passerine birds for nest boxes. However, relationships between hornets’ and birds’ occupancy rates may not be found^63^. In the Ore Mts., hornets were absent in nest boxes, and only wasps occupied one nest box during the autumn inspections. Thus, insects did not limit the boreal owl population in the Ore Mts. In the Trebon Basin, hornets only occupied 0–11% of our erected nest boxes during the spring inspections but up to 44–52% during autumn inspections. The period of nest box occupancy by insects did not overlap with that of the boreal owl in the Trebon Basin, avoiding interspecific competitions. Further, martens can raise their young in nest boxes and thus potentially compete for nesting sites with boreal owls. However, we discovered only three boxes with pine marten adults providing their young during spring inspections in the Ore Mts., and one such case was recorded in the Trebon Basin. These rare cases document that pine martens did not compete for nest boxes with boreal owls. Finally, passerine birds occupied up to 30% and 8% of erected boxes during spring inspections in the Ore Mts. and Trebon Basin, respectively. Since passerines are alternative prey for the boreal owl^3,18^, we are convinced that these birds would rather avoid the competition for nest boxes with the boreal owl. Overall, it seems clear that the competition with other animals was not a reason for the differences in nest box use between the study areas (the fourth hypothesis).

### Breeding performance in nest boxes

Martens depredated only 8% of nests in the Ore Mts., and one nest failed for the same reason in the Trebon Basin. The rate of nest predation by martens in nest boxes and natural cavities varies across regions. For example, in Germany, martens depredated 59% of owl nests placed in boxes, but only 24% in natural tree cavities^64^. In the Spain Pyrenees, the trend was the opposite; martens destroyed 50% of nests in tree cavities, but only 15% in nest boxes^65^. During the long-term study in the Ore Mts., we found out that the predation rate of boreal owl nests by martens varied hugely among years, reaching 0–50%; and the availability of *Apodemus* mice was its main drive^66^. In addition to nest failure due to predation, we also recorded that females abandoned 20–52% of nests in the Ore Mts., probably due to insufficient providing of females and nestlings by males^67^. Our study did not compare owl breeding success in nest boxes and tree cavities. However, Hruška^60^ performed long-term research (2006–2020, 123 nests) on a boreal owl population nesting in natural tree cavities in the Vysočina Hills in the central Czech Republic. Hruška^60^ found that 33–63% of boreal owl nests were successful each year, martens depredated 6–50% of nests, and up to 25% failed due to water flooding, including one nest where nestlings drowned (see also^68^). Water flooding does not happen in maintained nest boxes and has never been identified as a reason for nest failure in our study areas (Zárybnická, unpublished data). These findings indicate that breeding success of boreal owls nesting in wooden boxes and natural cavities is similar. Alternatively, maintaining and preventing nest boxes from undesirable environmental processes can increase owl breeding success in nest boxes compared to natural cavities.

### Recommendations for nest box deploying

We have documented that nest boxes can effectively support boreal owl populations and be a suitable tool for collecting biological information and material. At the same time, nest boxes can lose their supporting function for the target species under specific environmental conditions. Based on our findings, we provide the following specific recommendations for nest box deploying in Central European conditions to support boreal owl populations.

First, nest boxes should be distributed preferably in forest habitats that suffer from short-term or long-term lack of natural tree cavities. Examples of such habitats are young forest stands, intensively managed forest plantations, and forest stands exposed to other anthropogenic (e.g., atmospheric pollution), climatic (e.g., windthrow calamity, heavy snowfall), and biotic (e.g., an outbreak of insects or fungi) process followed by extensive forest losses. Such habitats suffer from the lack of black woodpeckers and other woodpeckers as well^3,4^. The size of the black woodpecker population can be a bioindicator of the availability of natural cavities for nocturnal boreal owls and a signal to provide additional nesting opportunities.

Second, nest boxes should be distributed preferably in coniferous forest habitats at high and middle elevations (above 600 m a. s. l.), providing optimal climate conditions for the study species^15,34,69^. Among owls in the Czech Republic, only the boreal owl prefers inhabiting the highest elevations^15,34^. Our findings support the elevational preference because the boreal owl was the most abundant owl species in all years on the Ore Mts. plateau. At the same time, the situation differed in the Trebon Basin lowlands, in which tawny owls dominated during two of three study years. Therefore, elevational optimum should be considered when practical support for the boreal owl is developed.

In general, during our study, we have identified a lack of specific recommendations for deploying nest boxes for the boreal owl. For example, it would be helpful to guide which habitats and environmental conditions to prefer for nest box deployment, which microhabitats to prefer for nest box installation, how nest boxes to maintain, when nest boxes to relocate, and which nest box dimensions and protection against predators to use. We conclude the comprehensive practical guides should be a topic for further review.

## Conclusions

The abundances and community structures of small mammals were similar in the Ore Mts. and Trebon Basin during the whole study, apart from one year (2017) when *Apodemus* mice peaked in the Ore Mts. Boreal owl populations were comparable in size based on territorial vocalisation in both study areas, but their readiness to occupy nest boxes differed substantially. Nest boxes were an efficient tool to support the availability of nesting opportunities for the study species in young restored forests in the historically air-polluted Ore Mts. However, nest boxes lost their supporting function for the boreal owl in mature Scots pine forests in the Trebon Basin. Therefore, deploying nest boxes should be considered carefully under local environmental conditions.

## Supplementary Information

Supplementary Information.

## Data Availability

All data produced from this study are available in the manuscript (Tables 1, 2, 3, 4, 5 and Figs. 1, 2, 3, 4) and the Supplementary Information file (Tables S1–S5).
