# Supplementary material for: Forest structure determines nest box use by Central European boreal owls
Source: Sci Rep. 2022 Mar 18;12:4735. doi: 10.1038/s41598-022-08792-y (PMC8933568; doi:10.1038/s41598-022-08792-y)
Supplement: Supplementary file 1 — Supplementary Information. [file 41598_2022_8792_MOESM1_ESM.pdf]

Supplementary Information

Forest structure determines nest box use by Central European boreal owls

Richard Ševčík, Bohuslav Kloubec, Jan Riegert, Jiří Šindelář, Marek Kouba & Markéta Zárybnická

Table S1. The abundance of small mammals in snap traps in the Ore Mountains and the Trebon Basin in 2015–2017. The number of individuals, trapping index (i.e., the number of individuals per 100 trap-nights, mean ± SD), and the total proportion of particular species are shown.

Study area	Species	2015<br>No. of inds.	 Trapping index	2016<br>No. of inds.	 Trapping index	2017<br>No. of inds.	 Trapping index	Total number	%
Ore Mts.	*Apodemus* mice	11	1.53 ± 1.11	0	0.00	30	4.17 ± 4.01	41	54.0
	Microtus voles	1	0.14 ± 0.34	0	0.00	1	0.14 ± 0.34	2	2.6
	Myodes voles	4	0.56 ± 0.68	1	0.14 ± 0.34	26	3.61 ± 6.53	31	40.8
	Sorex shrews	2	0.28 ± 0.68	0	0.00	0	0.00	2	2.6
	Total	18	2.50 ± 1.18	1	0.14 ± 0.34	57	7.92 ± 9.45	76	100.0
Trebon Basin	*Apodemus* mice	10	1.39 ± 1.88	2	0.28 ± 0.43	2	0.28 ± 0.43	14	53.9
	Microtus voles	1	0.14 ± 0.34	0	0.00	0	0.00	1	3.8
	Myodes voles	2	0.28 ± 0.68	4	0.56 ± 1.01	4	0.56 ± 0.86	10	38.5
	Sorex shrews	1	0.14 ± 0.34	0	0.00	0	0.00	1	3.8
	Total	14	1.94 ± 1.64	6	0.83 ± 1.39	6	0.83 ± 1.05	26	100.0

Table S2. The results of GLMM analysis (*lmer* function). The effect of the study area, year, and their interaction on the abundance of small mammals.

Model	AIC	df	Chi	% of explained variability	P
Apodemus mice					
null model	165.78	35			
null model + area	164.83	34	2.95	1.85	0.086
null model + year	163.58	33	3.25	3.71	0.071
null model + year*area	156.13	32	13.45	12.30	0.004
Myodes voles					
null model	181.94	35			
null model + area	182.83	34	1.11	0.63	0.292
null model + year	182.82	33	2.01	1.77	0.156
null model + year*area	184.53	32	4.29	4.21	0.232
Microtus voles					
null model	2.16	35			
null model + area	3.79	34	0.37	8.57	0.546
null model + year	3.91	33	1.89	36.95	0.170
null model + year*area	8.73	32	1.18	47.18	0.758
Sorex shrews					
null model	22.18	35			
null model + area	23.97	34	0.21	1.32	0.644
null model + year	22.19	33	3.78	24.70	0.052
null model + year*area	27.46	32	0.72	29.16	0.868

Table S3. Partial relationships of *Apodemus* mouse abundance between the study areas (Ore Mts., Trebon Basin) and years (2015–2017) using a post-hoc test (function *lsmeans* in lsmeans package).

Area	Year	Contrast	Estimate	df	t-ratio	P
Ore Mts.		2015 – 2016	1.53	20.0	1.54	1.000
Ore Mts.		2015 – 2017	-2.64	20.0	-2.66	0.134
Ore Mts.		2016 – 2017	-4.17	20.0	-4.21	0.004
Trebon Basin		2015 – 2016	1.11	20.0	1.12	1.000
Trebon Basin		2015 – 2017	1.11	20.0	1.12	1.000
Trebon Basin		2016 – 2017	0.00	20.0	0.00	1.000
	2015	Ore Mts. – Trebon Basin	0.14	28.4	0.13	1.000
	2016	Ore Mts. – Trebon Basin	-0.28	28.4	-0.26	1.000
	2017	Ore Mts. – Trebon Basin	3.89	28.4	3.58	0.011

Table S4. The number of sampling points, the number of sampling points with owl vocalisation, the vocal occupancy rate (i.e., the number of vocalising individuals per the number of sampling points), and the density of vocalising individuals related to the study area (Ore Mts., Trebon Basin), year (2015–2017), and period (April, May).

			Ore Mts.				Trebon Basin			
Year	Species	Month	No. of sampling points	No. of sampling points with vocalisation	Vocal occupancy rate	Density per 10 km^2	No. of sampling points	No. of sampling points with vocalisation	Vocal occupancy rate	Density per 10 km^2
2015	Boreal owl	April	34	20	0.59	1.87	31	16	0.52	1.64
		May	26	13	0.50	1.59	31	18	0.58	1.85
	Tawny owl	April	34	14	0.41	1.31	31	15	0.48	1.54
		May	26	10	0.38	1.22	31	15	0.48	1.54
	Eagle owl	April	34	0	0.00	0.00	31	2	0.06	0.21
		May	26	0	0.00	0.00	31	1	0.03	0.10
	Pygmy owl	April	34	3	0.09	0.28	31	6	0.19	0.62
		May	26	2	0.08	0.24	31	11	0.35	1.13
	Long-eared owl	April	34	3	0.09	0.28	31	1	0.03	0.10
		May	26	3	0.12	0.37	31	0	0.00	0.00
	Mean ± SD		30.0 ± 4.2	6.8 ± 6.9	0.23 ± 0.22	0.72 ± 0.70	31.0 ± 0.0	8.5 ± 7.2	0.27 ± 0.23	0.87 ± 0.74
2016	Boreal owl	April	36	17	0.47	1.50	31	7	0.23	0.72
		May	35	6	0.17	0.55	21	4	0.19	0.61
	Tawny owl	April	36	17	0.47	1.50	31	15	0.48	1.54
		May	35	14	0.40	1.27	21	8	0.38	1.21
	Eagle owl	April	36	1	0.03	0.09	31	0	0.00	0.00
		May	35	0	0.00	0.00	21	0	0.00	0.00
	Pygmy owl	April	36	2	0.06	0.18	31	9	0.29	0.92
		May	35	2	0.06	0.18	21	1	0.05	0.15

	Long-eared owl	April	36	3	0.08	0.27	31	2	0.06	0.21
		May	35	3	0.09	0.27	21	0	0.00	0.00
	Mean ± SD		35.5 ± 0.5	6.5 ± 6.8	0.18 ± 0.19	0.58 ± 0.60	26.0 ± 5.3	4.6 ± 5.0	0.17 ± 0.17	0.54 ± 0.55
2017	Boreal owl	April	36	18	0.50	1.59	32	11	0.34	1.09
		May	36	14	0.39	1.24	32	11	0.34	1.09
	Tawny owl	April	36	11	0.31	0.97	32	18	0.56	1.79
		May	36	9	0.25	0.80	32	14	0.44	1.39
	Eagle owl	April	36	0	0.00	0.00	32	1	0.03	0.10
		May	36	0	0.00	0.00	32	1	0.03	0.10
	Pygmy owl	April	36	1	0.03	0.09	32	9	0.28	0.90
		May	36	2	0.06	0.18	32	5	0.16	0.50
	Long-eared owl	April	36	5	0.14	0.44	32	3	0.09	0.30
		May	36	3	0.08	0.27	32	2	0.06	0.20
	Mean ± SD		36.0 ± 0.0	6.3 ± 6.4	0.18 ± 0.18	0.56 ± 0.56	32.0 ± 0.0	7.5 ± 6.0	0.23 ± 0.19	0.75 ± 0.59

Table S5. The number of boxes, the number of boxes occupied by animal taxa (boreal owls, common goldeneyes, pine martens, passerine birds, bats, and insects), and the nest box occupancy rate (the number of occupied nest boxes per available nest boxes) related to the study area (Ore Mts., Trebon Basin), year (2015–2017), and period (spring: April-May, autumn: September-October).

			Ore Mts.			Trebon Basin		
Year	Species	Period	Number of boxes	Number of occupied boxes	Nest box occupancy rate	Number of boxes	Number of occupied boxes	Nest box occupancy rate
2015	Boreal owl	Spring	230	23	0.10	245	3	0.01
	Passeriformes	Spring	230	11	0.05	245	20	0.08
	Insect	Spring	230	0	0.00	245	0	0.00
	Pine marten	Spring	230	3	0.01	245	0	0.00
	Goldeneye	Spring	230	0	0.00	245	0	0.00
	Bats	Spring	230	0	0.00	245	0	0.00
		Mean ± SD	230.0 ± 0.0	6.2 ± 9.3	0.03 ± 0.04	245.0 ± 0.0	3.8 ± 8.0	0.02 ± 0.03
2016	Boreal owl	Spring	246	23	0.09	242	2	< 0.01
	Passeriformes	Spring	246	74	0.30	242	17	0.07
	Insect	Spring	246	0	0.00	242	2	< 0.01
	Pine marten	Spring	246	2	< 0.01	242	1	< 0.01
	Goldeneye	Spring	246	0	0.00	242	0	0.00
	Bats	Spring	246	0	0.00	242	0	0.00
		Mean ± SD	246.0 ± 0.0	16.5 ± 29.6	0.07 ± 0.12	242.0 ± 0.0	3.7 ± 6.6	0.02 ± 0.03
	Boreal owl	Autumn	246	0	0.00	56	0	0.00
	Passeriformes	Autumn	246	19	0.08	56	0	0.00
	Insect	Autumn	246	1	< 0.01	56	29	0.52
	Pine marten	Autumn	246	0	0.00	56	0	0.00
	Goldeneye	Autumn	246	0	0.00	56	0	0.00
	Bats	Autumn	246	0	0.00	56	0	0.00

		Mean ± SD	246.0 ± 0.0	3.3 ± 7.7	0.01 ± 0.03	56.0 ± 0.0	4.8 ± 11.8	0.09 ± 0.21
2017	Boreal owl	Spring	246	18	0.07	237	0	0.00
	Passeriformes	Spring	246	49	0.20	237	19	0.08
	Insect	Spring	246	0	0.00	237	26	0.11
	Pine marten	Spring	246	0	0.00	237	0	0.00
	Goldeneye	Spring	246	0	0.00	237	1	< 0.01
	Bats	Spring	246	0	0.00	237	0	0.00
		Mean ± SD	246.0 ± 0.0	11.2 ± 19.9	0.05 ± 0.08	237.0 ± 0.0	7.7 ± 11.7	0.03 ± 0.05
	Boreal owl	Autumn	246	1	< 0.01	200	0	0.00
	Passeriformes	Autumn	246	15	0.06	200	38	0.19
	Insect	Autumn	246	0	0.00	200	87	0.44
	Pine marten	Autumn	246	0	0.00	200	0	0.00
	Goldeneye	Autumn	246	0	0.00	200	0	0.00
	Bats	Autumn	246	0	0.00	200	3	0.02
		Mean ± SD	246.0 ± 0.0	2.7 ± 6.1	0.01 ± 0.02	200.0 ± 0.0	21.3 ± 35.5	0.11 ± 0.18
